# Prevalence of Hypertension in Iran 1980–2012: A Systematic Review

**Published:** 2016-10-03

**Authors:** Masoud Mirzaei, Setareh Moayedallaie, Latife Jabbari, Masoud Mohammadi

**Affiliations:** 1 *Yazd Cardiovascular Research Center, Shahid Sadoughi University of Medical Sciences, Yazd, Iran.*; 2 *University of New South Wales, Sydney, Australia.*; 3 *Amin Hospital, Isfahan University of Medical Sciences, Isfahan, Iran.*; 4 *Department of Social Medicine, Kermanshah University of Medical Sciences, Kermanshah, Iran.*

**Keywords:** *Hypertension*, *Root cause analysis*, *Review literature as topic*, *Iran*

## Abstract

**Background: **The high prevalence of hypertension and concomitant increase in the risk of its related disease makes it an important health concern all over the world. Hypertension is one of the 5 global leading causes of mortality in the world. Little is known about the current prevalence of hypertension in Iran, however. This systematic review aimed to investigate the current prevalence of hypertension in Iran.

**Methods:** A systematic review of hypertension was conducted using international databases, including Medline (PubMed) and Science Direct (Scopus), and Persian scientific databases. The searched keywords were "hypertension", "raised blood pressure", "prevalence", and "Iran". All original articles in English published from 1980 to 2012 were included. After data extraction, heterogeneity between studies and publication bias was assessed and effect size was pooled by the random effect model.

**Results: **Forty-two studies with 402 282 subjects were included. The estimated prevalence of hypertension varied all around Iran (I^2 ^= 99%). The overall pooled prevalence of hypertension was 22% (95%CI: 20.2 - 23.8). The prevalence of hypertension was 23.6% (95%CI: 21.1 - 26.1) in men and 23.5% (95%CI: 20.2 - 23.8) in women. In urban areas, the prevalence of hypertension was 22.1% (95%CI: 19.4 - 24.7). Ten studies investigated the prevalence of hypertension in rural areas and according to the random effect model, the prevalence of hypertension in rural areas was 18.6% (95%CI: 13.6 - 23.6). Nonsignificant publication bias was found in this review (p value = 0.18). In our meta-regression analysis, only mean age and study quality were associated with significant variability.

**Conclusion: **According to this study, hypertension is one of the most common health problems in Iran. Around one-quarter of the adult population is hypertensive and its prevalence increases by aging. Timely and appropriate public health strategies are essential for the improvement of the screening, treatment, and control of hypertension.

## Introduction

Numerous epidemiological studies have shown the association between hypertension and cardiovascular events in low-income and high-income countries.^1^^-^^3^ The prevalence of hypertension ranges from 15% to 37% of the total population in different areas of the world.^4^^-^^6^

In those 2 countries and other low-income countries, the average age of hypertensive patients is lower than that in high-income countries.^4^

Low-income countries bear about two-thirds of the total burden of hypertension in the world, with China and India having the highest prevalence rate of hypertension in the world.^5^

The results of prospective studies in Asia with more than 500 000 cases have shown a direct relation between normal levels of systolic and diastolic blood pressures and the risk of coronary heart disease and stroke in both white and Asian populations.^2^^-^^5^

The WHO estimates that 600 million people around the world are at risk of major cardiovascular events, including myocardial infarction, stroke, and heart failure, due to high blood pressure. Furthermore, hypertension accounts for 13% of all mortality (about 7.1 million deaths per year), 62% of all stroke, and 49% of all myocardial infarction.^7^^, ^^8^

The reduction in the number of hypertensive patients in Western countries during the past 30 - 40 years has been attributed to a high level of awareness and control of known risk factors.^9^

There are only a few studies on the overall prevalence of hypertension in developing countries, including Iran. A systematic review on hypertension in Iran reported data from a decade ago; however, it did not report the prevalence in rural and urban areas and included very few papers.^10^ The present systematic review aimed to investigate the prevalence of hypertension in Iran during the period of 1980 - 2012. The result can be a basis for future research and health planning.

## Methods

Persian scientific databases, including IranMedex and Scientific Information Database (SID), and international databases, including Medline (PubMed) and ScienceDirect (Scopus), were searched to find relevant papers. The searched keywords were "hypertension", "raised blood pressure", "prevalence", and "Iran" in English and Persian. All original studies in English published from 1980 to 2012 which met our quality criteria were included.

**Figure 1:**
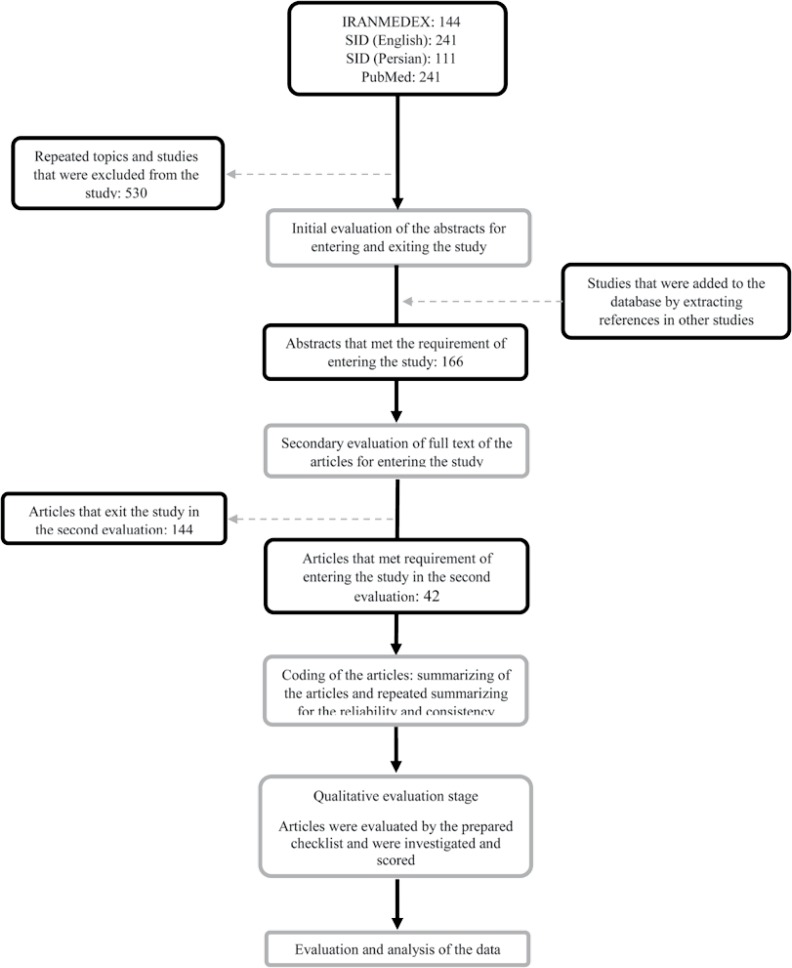
Flow chart of the included papers.

At the next step, the "citation pearl growing" technique^11^ was used and the bibliography of each extracted paper was searched manually. Abstracts from the searched articles were initially investigated, and first screening was performed considering the titles and abstracts. Articles were reviewed if they were peer-reviewed and reported hypertension and its prevalence in an Iranian population. Subsequently, full texts of the articles were extracted and if their data sources were similar, the most detailed article was selected. In the case of unavailability of full-text articles, the authors were contacted to provide the full text. Figure 1 shows the details of the process of selecting and excluding papers.

The selection criteria for the included papers were: a) population-based studies which reported the prevalence of hypertension, b) age of the population groups ≥ 15 years, c) using random or total (census) sampling of the studies, and d) using standard methods to measure hypertension. The criteria for hypertension were based on the 7th Joint National Committee on Prevention, Detection, Evaluation, and Treatment report (JNC7), which were systolic blood pressure > 140 mm Hg and diastolic blood pressure > 90 mm Hg or self-reports of those taking medication for hypertension.^10^

Studies that met the criteria to enter the review were extracted and coded in FileMaker Pro (version 8.0). A number of studies (10%) were summarized and re-evaluated to check the consistency of data extraction. The studies were evaluated using a checklist for their content and quality. The checklist was based on critical appraisal criteria for the health research literature published by Loney et al.^12^ Questions regarding the design of the study included the use of appropriate method and sampling frame, sample size, response rate, and appropriate interpretation of the data according to the objective of the study.

The first step in data analysis was to determine the estimated prevalence of hypertension in all included studies. The heterogeneity test (Q test) was performed to determine whether the difference in prevalence estimates across the studies was more than chance. Furthermore, the Egger test was employed to investigate the probability of interpretation bias. Because there was a significant heterogeneity across the study results, a meta-regression analysis of single changing was performed. The effects of variables such as methodology, geographical area of the study (i.e., urban and rural), and mean age of the study population on the prevalence estimate were investigated. This strategy investigated which variables, including the sample size, publication year, and quality score of the article, affected the final results: none of them changed the results significantly. All stages of the statistical analyses were done using Kruskal–Wallis and random effect model in Stata/SE (version 10). 

Totally, 496 papers were identified, and 166 articles met the criteria for entering the study. The full text of the articles was further reviewed, and 118 articles were entered into the review (Figure 1). 

## Results

Of a total of 118 articles, 55 reported the prevalence of hypertension according to the JNC7 criteria. Four articles reported special population groups,^13^^-^^16^ and 6 papers reported hypertension according to separated systolic and diastolic pressures,^17^^-^^22^ which were excluded from the analysis.

Five articles were from the Healthy Heart Project in Isfahan.^23^^-^^27^ To avoid duplication due to multiple subjects used, we extracted data from the most completed report.^23^

Two studies investigated the prevalence of hypertension in more than 1 province.^24^^, ^^28^ One study investigated the trend of hypertension in populations > 18 years old in Isfahan over a 10-year period (1991 - 2001) in 3 cross sections. In these studies, every sub-study was considered an independent study.

Overall, 41 studies^29^^-^^69^ met the inclusion criteria published in the period of 1994 – 2010 (Table 1).

**Figure 2:**
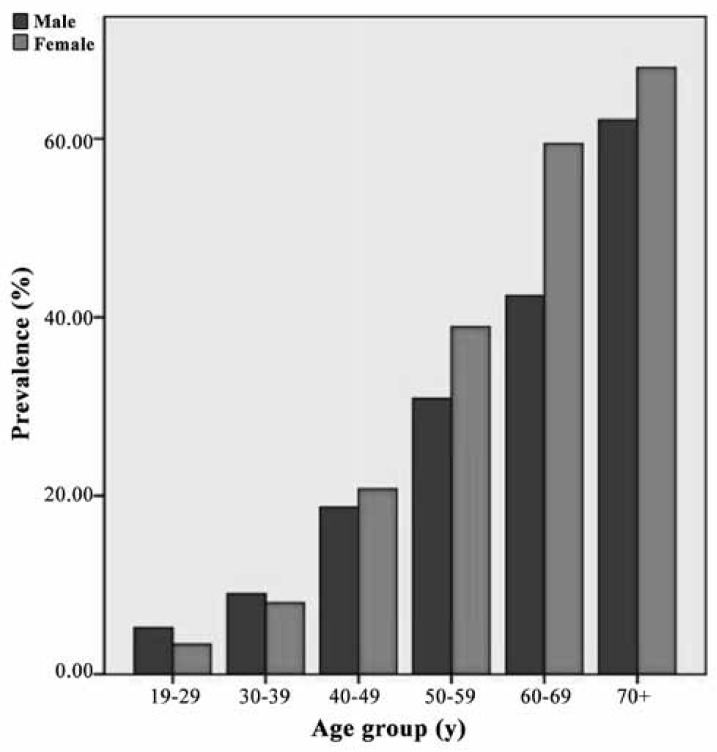
Variations in the prevalence of hypertension by age groups in males and females in Iran (1980–2012).

Figure 2 depicts the variations in the prevalence of hypertension by age groups in males and females in Iran over the period of study. From the age of 40, hypertension was more prevalent in women.

**Figure 3:**
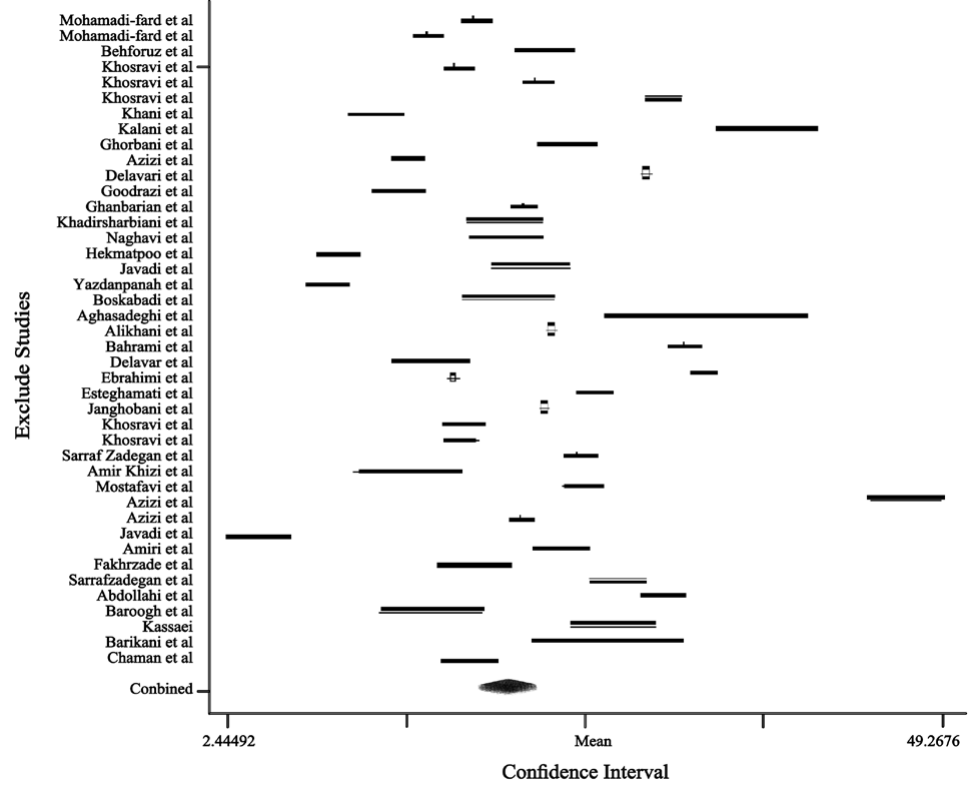
Forest plot of the included papers on high blood pressure in Iran 1980-2012.

Only in 8 studies was the prevalence of hypertension reported in similar age groups. According to our statistical analyses, there was a significant relationship between age groups and hypertension (p value < 0.001). Figure 3 illustrate a direct relationship between age and hypertension in both sexes.

**Figure 4:**
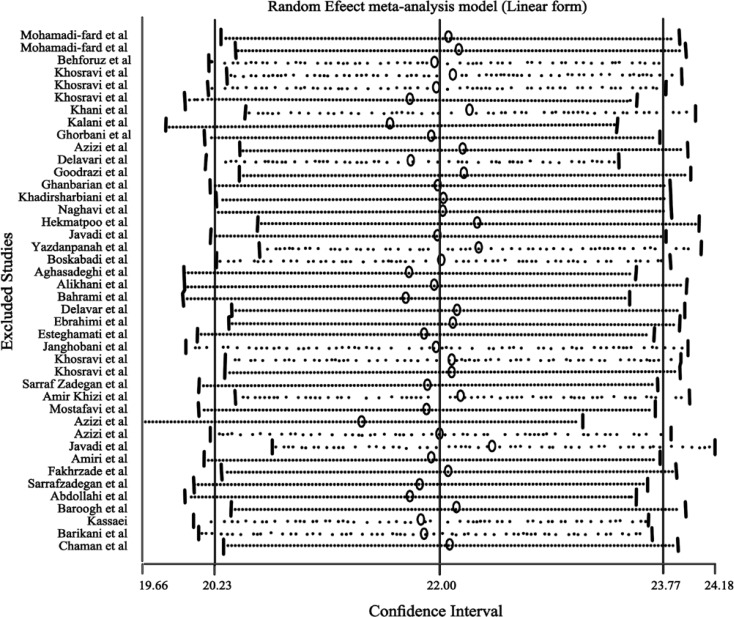
Influence analysis on the effect of the excluded papers on the final estimates of the prevalence of hypertension in Iran.

In 23 studies, the prevalence of hypertension was separated by gender and 4 studies were only done on women. The prevalence of hypertension was 23.5% (95%CI: 20.2 - 23.8) in women and 23.6% (95%CI: 21.1 - 26.1) in men. With the use of the random effect model and according to our meta-regression analysis, in 23 studies that investigated hypertension in men and women, we found no significant difference between gender and hypertension. Influence analysis revealed that ignoring any of the included studies did not change the overall estimate (Figure 4).

Thirty-one studies investigated hypertension in urban areas, and there was a significant heterogeneity among them. The overall prevalence of hypertension was 22.1% (95%CI: 19.4 - 24.7) in urban areas. Ten studies investigated the prevalence of hypertension in rural areas and according to the random effect model, the prevalence of hypertension in rural areas was 18.6% (95%CI: 13.6 - 23.6).

**Table 1:** Full description of the included articles published on hypertension in Iran (1980–2002).

Authors	Year	City	province	Urban/Rural	Sample Size	Age range (y)	sex	Prevalence (%)	Quality score
Mohamadi-Far et al.	2003	Arak	Arak	U/A	6175	Over 19	M/F	15.7	11
Mohamadi-Fard et al.	2003	Isfahan	Isfahan	U/A	6339	Over 19	M/F	18.8	11
Behforuz et al.	2002	Rafsanjan	Kerman	U/A	2000	Over 18	M/F	23.4	11
Khosravi et al.	1994	Isfahan	Isfahan	U	6000	Over 18	M/F	31.2	11
Khosravi et al.	1998	Isfahan	Isfahan	U	6781	Over 18	M/F	22.8	11
Khosravi et al.	2001	Isfahan	Isfahan	U	8104	Over 18	M/F	17.5	11
Khani et al.	2002	Tarom	Zanjan	R	1500	Over 15	M/F	12.3	8
Kalani et al.	2001	Yazd	Yazd	U	1129	Over 18	M/F	38.1	10
Ghorbani et al.	2008	Semnan	Semnan	U/A	3799	30-69	M/F	24.3	9
Azizi et al.	2008	Kermanshah	Kermanshah	U	4713	Over 15	M/F	14.3	7
Delavari et al.	2007	Iran	Iran	U	75132	Over 20	M/F	30.1	15
Goodarzi et al.	2003	Zabol	Sistan Baloochestan	U	1530	Over 18	M/F	13.9	9
Khadirsharbiani et al.	2001	Arak	Arak	U	1050	Over 20	M/F	20.7	4
Naghavi et al.	2000	Gonabad	S. Khorasan	U	1259	Over 18	M/F	20.9	8
Hekmatpoor et al.	1998	Arak	Markazi	U	2000	Over 20	M/F	9.6	7
Javadi et al.	2008	Ghazvin	Ghazvin	U	1000	Over 20	M/F	22.4	11
Yazdanpanah et al.	1998	Sanandaj	Kordestan	U	1722	Over 10	M/F	9.0	7
Boskabadi et al.	2006	Mashahd	R. Khorasan	U	704	Over 18	M/F	20.9	10
Aghasadeghi et al.	2008	Lorestan	Lorestan	Nomads	216	21-80	M/F	34.3	3
Alikhani et al.	2009	Iran	Iran	U/A	70981	25-64	M/F	23.8	15
Bahrami et al.	2006	Golestan	Golestan	U/A	8998	35-81	M/F	32.5	10
Delavar et al.	2009	Babol	Mazandaran	U	984	30-50	F	15.9	8
Ebrahimi et al.	2006	Iran	Iran	U/A	29972	15-64	M/F	17.4	12
Esteghamati et al.	2009	Iran	Iran	U/A	5287	15-64	M/F	26.6	12
Janghorbani et al.	2008	Iran	Iran	U/A	89404	25-65	M/F	18.2	13
Khosravi et al.	2009	Arak	Markazi	U/A	4853	Over 19	M/F	17.9	11
Khosravi et al.	2009	Isfahan	Isfahan	U/A	4719	Over 19	M/F	17.8	11
Sarrafzadegan et al.	1997	Isfahan	Isfahan	U/A	8639	19-70	M/F	25.5	8
AmirKhizi et al.	2008	Kerman	Kerman	R	370	20-45	F	14.3	7
Mostafavi et al.	2002	Shiraz	Fars	U	4045	Over 13	M/F	25.8	11
Azizi et al.	2003	Tehran	Tehran	U	1766	Over 60	M/F	46.9	10
Azizi et al.	2004	Teharn	Tehran	U	8647	20-70	M/F	21.7	13
Javadi et al.	2009	Minoodar	Ghazvin	U	400	Over 20	F	4.5	7
Amiri et al.	2004	Bushehr	Bushehr	U	2092	Over 25	M/F	24.5	6
Fakhrzade et al.	2001	Bushehr	Bushehr	U	1036	30-64	M/F	18.9	6
Sarrafzadegan et al.	1999	Isfahan	Isfahan	U	2200	19-70	M/F	28.1	10
Abdollahi et al.	2007	Golestan	Golestan	U	5000	17-70	M/F	31.0	11
Baroogh et al.	2010	Ghazvin	Ghazvin	U	450	24-65	M/F	15.8	8
Kassaei et al.	2010	Zanjan	Zanjan	U/A	1000	15-67	M/F	27.8	8
Barikani et al.	2010	Minoodar	Ghazvin	U	328	Over 30	F	27.2	8
Chaman et al.	2008	Agh Ghala	Golestan	R	1500	Over 30	M/F	18.4	7

U/A, Unavailable; U, Urban; R, Rural

Three national surveys were performed to evaluate risk factors for noncommunicable diseases from 2004 to 2006.^4^^, ^^6^ The overall prevalence of hypertension was reported to be 30.1% in 2004, 17.4% in 2005, and 26.6% in 2006. Another study investigated the trend of hypertension over a decade in Isfahan. According to 3 measurements, the prevalence rates were 31.2% in 1992, 22.8% in 1998, and 17.5% in 2001.

## Discussion

Overall, the prevalence of hypertension in Iran was 22% over the period, with the figure being close to those reported by other Middle Eastern countries.^28^ the prevalence was almost the same between men and women. However, it was higher in urban areas than in rural areas. The prevalence of hypertension in Iran was lower than that reported from the prevalence was higher than that in South Korea (19.8%),^28^ US (28%)^64^, China (27%)^65^ and the Nonetheless and several Arab countries of the Middle East (26.1%–32.2%),^66^^, ^^67^ Cameroon (15.4%).^68^

There are contradicting results regarding the relationship between gender and hypertension in developed countries. Unlike the result of this study and those of Haghdoost et al.,^69^ the prevalence of hypertension is higher in men than in women in Iran.^65^ In Saudi Arabia, hypertension in men (27.1%) is significantly higher than that in women (23.9%), whereas in the US and the Netherlands, hypertension is reportedly higher in women than in men.^65^ In Yemen, as a low-income country, hypertension is also slightly higher in women than in men (14.8% vs. 14.2%, respectively).^29^ Nevertheless, according to a previous systematic review of hypertension in Iran, there was a significant difference (1.3%) in the prevalence of hypertension between men and women.^29^^, ^^69^

In a study on hypertension in different age and sex groups, men < 40 years old had a higher prevalence of hypertension than women; this finding is line with ours. However, in older age groups, the prevalence of hypertension was higher in women than in men. Findings from studies in Latin America and Caribbean, Sub-Saharan Africa, and Slovakia have also demonstrated similar results.^65^ In a systematic review in Sub-Saharan Africa, the prevalence of hypertension was higher in the urban population than in the rural population.^69^ A national study in China in 2000 – 2001 also showed that hypertension in all age groups was higher in urban areas than in rural areas.^30^ In South Korea, however, the prevalence of hypertension was not significantly different between the urban and rural populations.^28^ Furthermore, a large study in Thailand on a population > 35 years of age showed a significant difference in the prevalence of hypertension between urban (26%) and rural areas (18%).^31^ Different lifestyle risk factors such as obesity, higher intake of salt and processed foods, and sedentary lifestyle can explain the higher prevalence of hypertension in urban areas.^69^^, ^^32^

There are some limitations to this study. First, there was disagreement between the reported prevalence of hypertension of the same area in different studies. Part of the differences could be explained by the different age range of the participants. In some studies, the adult population was considered > 15 years old, while in the others it was defined as a population > 20 years old. Second, the quality of the studies was different. On the basis of our meta-regression analysis, there was a significant relationship between the prevalence of hypertension and the quality score of the studies. In the high-quality studies, the reported prevalence of hypertension was higher than that of the medium-quality studies. Third, by careful searching of the Persian scientific databases, this study aimed to include all the related articles published in Iran over the period. However, full texts of a few studies were not available and detailed information was not reported in several articles. Although we contacted some of the authors for detail, we were unable to obtain data in some instances. Fourth, the prevalence of hypertension by standard age groups was reported in only a few studies, rendering it difficult for the authors to compare the prevalence across different age groups. 

## Conclusion

The prevalence of hypertension in Iran was close to the highest figure reported in the Middle East. This study revealed the need for a regular survey of hypertension utilizing a standard methodology. Future research with standard age groups, a standard definition of hypertension, and a valid research design should be conducted to compare the findings across various studies in different areas and time.

## Notes:

***This paper should be cited as:*** Mirzaei M, Moayedallaie S, Jabbary L, Mohammadi M. Prevalence of Hypertension in Iran 1980–2012: A Systematic Review. J Teh Univ Heart Ctr 2016;11(4):159-167.
